# AI-Assisted Dynamic Postural Control Screening to Improve Functional Mobility in Older Adult Populations: Quasi-Experimental Study

**DOI:** 10.2196/73290

**Published:** 2025-11-28

**Authors:** Kai-Chih Lin, Rong-Jong Wai, Hung-Yu Chang Chien

**Affiliations:** 1 Department of Electronic and Computer Engineering, National Taiwan University of Science and Technology, No. 43, Sec. 4, Keelung Rd., Da'an Dist., Taipei, 106335, Taiwan, 886 935636806, 886 227376367; 2 Department of Health Promotion and Health Education, National Taiwan Normal University, Taipei, Taiwan

**Keywords:** artificial Intelligence, fall risk assessment, dynamic postural control, older adult mobility, older adult fall prevention

## Abstract

**Background:**

Falls are a major cause of disability among older adults, and early identification of functional decline is essential for prevention. Artificial intelligence (AI) systems may enhance mobility screening by providing objective, real-time feedback.

**Objective:**

This study aimed to evaluate whether AI-assisted dynamic postural control screening combined with adaptive training improves functional mobility outcomes in older adult populations.

**Methods:**

A quasi-experimental study was conducted with 2005 older adults recruited from community centers and health care institutions in Keelung, Taiwan. Participants were assigned to either an experimental group (n=1451), which underwent AI-assisted screening with adaptive exercise prescriptions, or a control group (n=554), which completed follow-ups through regular physical assessments with standard care without AI-tailored training. The AI system integrated skeletal tracking with the Short Physical Performance Battery to assess balance, gait speed (4-m walk), and sit-to-stand performance. Independent-samples 2-tailed *t* tests and repeated-measures ANOVA were applied, and effect sizes (Cohen *d* and η²) with 95% CIs were reported.

**Results:**

The experimental group demonstrated significantly greater improvements compared with the control group in Short Physical Performance Battery scores (Δ=0.8 vs 0.3; *t*_2003_=3.41; *P*=.001; Cohen *d*=0.45, 95% CI 0.18‐0.72), gait speed (Δ=15 cm/s vs 5 cm/s; *t*_2003_=4.85; *P*<.001; Cohen *d*=0.62, 95% CI 0.35‐0.88), and sit-to-stand time (Δ=–1.4 s vs –0.6 s; *t*_2003_=3.12; *P*=.002; Cohen *d*=0.39, 95% CI 0.12‐0.65). Here “Δ” refers to the change score, calculated as post-intervention minus baseline (ie, the amount of improvement during the study period). Participation rate was strongly associated with outcomes, with 1-way ANOVA showing significant group differences (*F*_2,1448_=8.74‐12.21; *P*<.001; η²=0.07‐0.10).

**Conclusions:**

AI-assisted dynamic postural control screening combined with adaptive training substantially improved functional performance in mobility, balance, and gait among older adults. While fall incidence was not directly measured, these functional gains may have implications for fall risk reduction. Future longitudinal studies with extended follow-up (12‐24 mo) and prospective fall incidence tracking across diverse populations are required to validate whether these improvements translate into actual reductions in fall risk.

## Introduction

### Background on Fall Risks in the Older Adult Population

Falls are one of the leading causes of disability, hospitalization, and mortality among older adults [1,2]. Early detection of functional decline is critical for fall prevention; however, conventional assessments such as the Short Physical Performance Battery (SPPB) rely heavily on manual observation, which is time-consuming and subject to interrater variability [3,4]. With the rapid advancement of digital health technologies, artificial intelligence (AI) has emerged as a promising tool for objective, real-time mobility screening [5-7].

Recent studies have demonstrated that AI-based motion analysis can improve the accuracy and scalability of functional assessments in older adult populations [3,6,8-10]. Unlike static paper-based tests, AI can dynamically track skeletal movement, quantify gait and balance parameters, and provide continuous feedback. These innovations enable early detection of mobility decline and offer opportunities for personalized interventions [5,7,9-11]. Despite these advances, limited large-scale evidence exists regarding whether AI-assisted screening, when combined with adaptive training, leads to measurable improvements in functional mobility outcomes among older adults.

Recent *JMIR* studies have also highlighted the feasibility of integrating SPPB with broader fall risk assessment tools [12], the application of explainable AI for fall prediction [13], and the potential of digital and telemedicine-based prevention programs [14,15]. Moreover, wearable technology is increasingly considered a viable option for health care monitoring [16].

### Research Objectives and Contributions

This study investigates the effectiveness of AI-assisted dynamic postural control screening combined with adaptive training in improving mobility outcomes among older adults. The research makes three key contributions:

1. Demonstrating the feasibility of integrating AI skeletal tracking with the standardized SPPB to objectively assess gait speed, balance, and sit-to-stand performance.
2. Evaluating the short-term functional improvements attributable to AI-generated personalized training prescriptions.
3. Providing large-scale evidence on the relationship between participation rate and functional gains in an older adult population.

### Research Question

This study addressed the following research question: can AI-assisted dynamic postural control screening combined with adaptive training significantly improve functional mobility outcomes (SPPB score, gait speed, and sit-to-stand performance) compared with standard care in older adult populations?

### Hypotheses

This study had the following research hypotheses:

1. Participants in the experimental group will demonstrate significantly greater improvements in SPPB scores compared with the control group.
2. Participants in the experimental group will show significantly faster improvements in gait speed and sit-to-stand time compared with the control group.

While prior studies have demonstrated that the SPPB is a reliable predictor of functional decline [12,13], its traditional administration is limited by subjectivity and reliance on manual timing or scoring [14]. AI-assisted motion analysis offers a scalable solution by automating the assessment process, increasing measurement precision, and enabling real-time feedback [15,16].

This study builds upon existing evidence by integrating AI-based skeletal tracking with SPPB assessments and extending the application to a large older adult cohort. The primary aim is not only to demonstrate feasibility but also to evaluate whether AI-assisted screening, when combined with adaptive training prescriptions, leads to measurable improvements in mobility outcomes. Imran et al [10] and Santos et al [11] specifically support the dual rationale of integrating AI into both functional assessment and individualized prescription, underscoring the novelty of this approach.

By conducting a quasi-experimental study with more than 2000 participants, this research provides large-scale evidence on the effectiveness of AI-assisted interventions. Unlike prior work that focused mainly on prediction or assessment, our approach tested whether real-time AI-driven prescriptions translated into tangible improvements in balance, gait, and sit-to-stand performance.

## Methods

### Study Design and Participants

This quasi-experimental study was conducted in Keelung, Taiwan, over a 6-month period. A total of 2005 older adults (≥65 years) were recruited through stratified sampling from 12 community centers and 9 health care institutions.

The experimental group (n=1451) was recruited primarily from community centers. Participants underwent AI-assisted SPPB assessments and received individualized exercise prescriptions dynamically adapted by the AI system. Prescriptions were adjusted at subsequent assessments according to participants’ performance.

The control group (n=554) was recruited from health care institutions. Participants underwent AI-assisted assessments and continued with standard care (ie, routine health advice and monitoring by their institutions) but did not receive AI-tailored prescriptions. This ensured ethical parity while isolating the effect of AI-enabled personalization.

**Figure 1.**
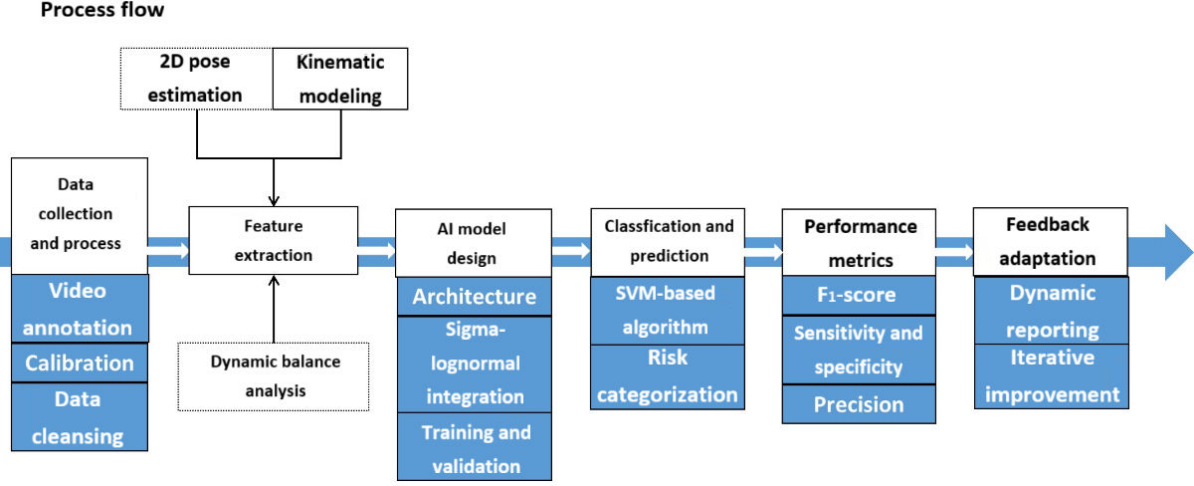
Flow diagram of participant recruitment, group allocation, intervention procedures, and follow-up assessments. AI: artificial intelligence; SVM: support vector machine.

Importantly, baseline differences reflected recruitment sources: the experimental group had a mean SPPB score of 11.2 (SD 1.8), whereas the control group had a mean score of 10.4 (SD 1.8). These differences were expected, given the healthier status of community-dwelling participants compared with clinical recruits. Independent-sample tests confirmed that baseline differences did not bias the interpretation of intervention outcomes. A summary of participant recruitment, group allocation, and follow-up assessments is presented in Figure 1.

### Sample Size and Power Analysis

Although not prespecified, a post hoc power analysis using G*Power (Heinrich Heine University Düsseldorf) indicated that a minimum of 788 participants per group would be required to detect a medium effect size (Cohen *d*=0.4) with 90% power at *α*=.05. Our actual sample sizes (1451 and 554) exceeded this requirement, ensuring sufficient statistical power and confirming that the large cohort was methodologically and ethically justified.

### Assessment Tools and AI Integration

Functional mobility was assessed using the SPPB, which is widely validated as a predictor of functional decline and fall risk [4,11-13]. Wearable and sensor-based technologies further demonstrate feasibility for continuous monitoring and objective evaluation [16]. The SPPB included (1) balance tests, (2) 4-meter gait speed, and (3) the 5-times sit-to-stand test. Assessments were conducted with AI-assisted skeletal tracking to improve accuracy and objectivity.

The AI system replaced traditional manual scoring by extracting quantitative motion features in real time. This consolidated statement replaces prior repeated descriptions, improving readability while preserving scientific accuracy. Key features included the following:

- Real-time monitoring: continuous capture of postural stability, gait patterns, and transitional movements.
- Automated data processing: machine learning algorithms compared participant movements against validated mobility benchmarks.
- Immediate feedback: results were processed instantly, producing individualized reports for trainers and health care staff.

### System Validation

**Figure 2.**
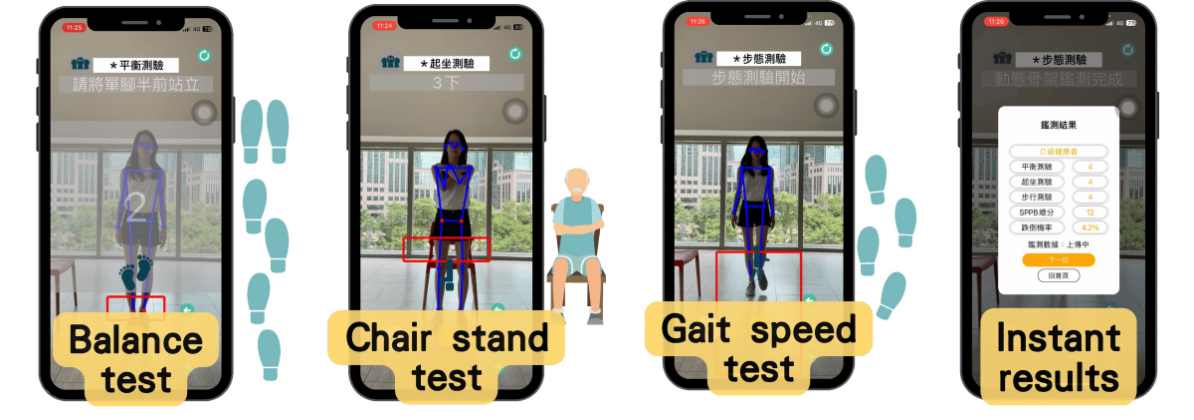
Architecture and workflow of the artificial intelligence–assisted skeletal tracking system, including video capture, landmark extraction, feature quantification, and automatic scoring with feedback.

In a prior pilot study of 320 older adults, the AI algorithm achieved greater than 90% accuracy in classifying functional mobility categories compared with physiotherapist ratings. Test-retest reliability was high (intraclass correlation coefficient >0.88) for gait speed and sit-to-stand measures, supporting the robustness of the system. The architecture and workflow of the AI system are summarized in Figure 2, showing the process from skeletal tracking and feature extraction to automatic scoring and feedback generation.

### Ethical Considerations

This study was approved by the Institutional Review Board of En Chu Kong Hospital (ECKIRB1071204). All participants were informed about the study objectives and procedures and provided written informed consent before enrollment. No financial incentives or compensation were provided for participation. All data were anonymized, and confidentiality and data security measures were strictly implemented to protect participant privacy in accordance with institutional and national ethical guidelines.

### AI-Enabled Training Adaptation

In the experimental group, the AI system generated individualized exercise prescriptions that were adapted at subsequent assessments. Algorithms adjusted the type, intensity, and progression of exercises based on gait speed thresholds, sit-to-stand performance, and balance stability metrics.

**Figure 3.**
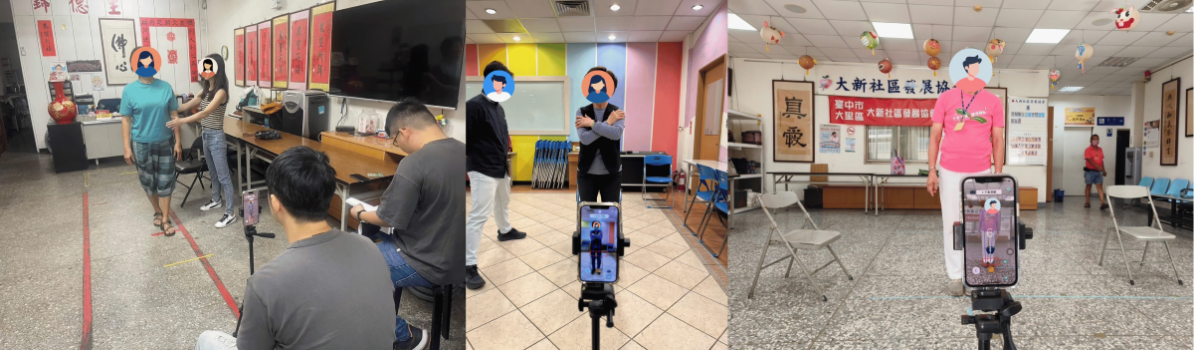
Framework of artificial intelligence–enabled adaptive training prescriptions, showing how balance, gait speed, and sit-to-stand performance metrics informed real-time adjustments to exercise type, intensity, and progression.

This ensured tailored interventions beyond generic training exposure. The control group used the AI system solely for assessment, without adaptive prescriptions. The adaptive prescription framework is presented in Figure 3, which illustrates how SPPB-derived performance metrics (ie, balance, gait, and sit-to-stand performance) informed AI-driven adjustments in exercise type, intensity, and progression.

### Data Collection Procedures

Participants in both groups were assessed at baseline and 6-month follow-up. Monthly interim assessments were conducted to monitor progress. All assessments were video recorded through the AI skeletal tracking system and processed automatically.

Data collected included balance scores from SPPB balance tasks; gait speed (cm/s) from the 4-meter walk; sit-to-stand time (s) from the 5-repetition test; and demographics such as age, sex, and recruitment source.

AI-generated feedback reports were shared with trainers to guide intervention adjustments in the experimental group.

### Statistical Analysis

All statistical analyses were performed using SPSS (version 27.0; IBM Corp). Descriptive statistics (means and SDs) were calculated for all measures. Between-group comparisons were conducted using independent-samples *t* tests to compare improvements in SPPB, gait speed, and sit-to-stand performance between the experimental and control groups. Within-group changes were examined using repeated-measures ANOVA to examine changes from baseline to follow-up within each group. Participation-level analysis was performed using one-way ANOVA to compare outcomes among subgroups with high (>75%), moderate (50%‐75%), and low (<50%) participation rates, to explore the relationship between adherence and functional outcomes. Effect sizes were calculated using Cohen *d* for pairwise comparisons, and η² was reported for ANOVA models; 95% CIs were reported for all effect size estimates. Statistical significance was set at a 2-tailed α=.05.

## Results

### Baseline Characteristics

A total of 2005 participants were analyzed, including 1451 in the experimental group and 554 in the control group. The experimental group had a mean age of 73.4 (SD 6.1) years, with 841 (57.9%) female participants. The control group had a mean age of 74.1 (SD 6.4) years, with 338 (61.0%) female participants.

Baseline differences in walking speed were noted. As shown in Table 1, the experimental group demonstrated a mean baseline gait speed of 117 cm/s, compared with 105 cm/s in the control group. These values align with cut-off thresholds for functional decline in mobility. Baseline functional measures showed expected differences due to recruitment sources (community vs health care institutions):

- Experimental group: SPPB**=**11.2, gait speed=117 cm/s, and sit-to-stand=10.5 seconds.
- Control group: SPPB**=**10.4, gait speed=105 cm/s, and sit-to-stand=11.2 seconds.

An SPPB score of **≤**9 was considered indicative of high fall risk, while scores ≥10 represented normal to moderate function, consistent with prior validation studies. Independent-samples tests confirmed that although baseline functional status differed, these differences did not bias the interpretation of intervention outcomes.

**Table 1.** Comparison of artificial intelligence–assisted and Standard Short Physical Performance Battery (SPPB) Results.

Metric	Experimental group (baseline), mean (SD 1.8)	Experimental group (end), mean (SD 1.6)	Control group (baseline), mean (SD 1.8)	Control group (end), mean (SD 1.7)	Improvement difference	*P* value	Cohen *d*
SPPB score	11.2	12.0	10.4	10.7	0.5	.008	0.45
Gait speed (cm/s)	117	132	105	110	10	<.001	0.62
Sit-to-stand (s)	10.5	9.1	11.2	10.6	–0.8	.006	0.39

### Intervention Outcomes

#### Overview

The improvements in SPPB, gait speed, and sit-to-stand performance observed in this study are consistent with findings from prior comparative validation studies using SPPB and Grupo de Desenvolvimento Latino-Americano para a Maturidade batteries [6,17]. Our AI-assisted skeletal tracking showed high accuracy and reliability, similar to other automated approaches such as camera-based SPPB evaluation [18] and sensor insole–based gait analysis [19].

#### SPPB Scores

The experimental group’s mean SPPB score increased from 11.2 to 12.0 (Δ=0.8), while the control group’s mean SPPB score increased from 10.4 to 10.7 (Δ=0.3). The improvement difference was 0.5 points (*t*_2003_=3.41; *P*=.001; Cohen *d*=0.45, 95% CI 0.18-0.72).

#### Gait Speed

The experimental group’s gait speed improved from 117 cm/s to 132 cm/s (Δ=15 cm/s), compared with the control group’s improvement from 105 cm/s to 110 cm/s (Δ=5 cm/s). The improvement difference was 10 cm/s (*t*_2003_=4.85; *P*<.001; Cohen *d*=0.62, 95% CI 0.35-0.88).

#### Sit-to-Stand Performance

The experimental group reduced their sit-to-stand time from 10.5 s to 9.1 s (Δ=–1.4 s), compared with the control group’s reduction from 11.2 s to 10.6 s (Δ=–0.6 s). The improvement difference was **–**0.8 s (*t*_2003_=3.12; *P*=.002; Cohen *d*=0.39, 95% CI 0.12-0.65).

### Participation-Level Analysis

Participation rates strongly influenced outcomes [17]. The differences across participation levels are summarized in Table 2. The experimental group was divided into high (>75%), moderate (50%‐75%), and low (<50%) attendance subgroups.

One-way ANOVA confirmed significant differences across participation levels for all three functional outcomes. As shown in Table 2, higher participation was associated with greater improvements in SPPB scores, gait speed, and sit-to-stand performance.

**Table 2.** Summary of participation impact with statistical tests.

Metric	High participation (>75%; n=482), n (%)	Moderate participation (50%‐75%; n=515), n (%)	Low participation (<50%; n=454), n (%)	*F* test (*df*)	*P* value	η²
SPPB^a^ score (Δ)	1.2 (10.0)	0.8 (7.0)	0.3 (2.0)	8.74 (2, 1448)	<.001	0.07
Gait speed (Δ, cm/s)	20 (17.0)	12 (10.0)	5 (4.0)	12.21 (2, 1448)	<.001	0.10
Sit-to-stand (Δ, s)	–1.8 (15.0)	–1.0 (9.0)	–0.4 (4.0)	9.63 (2, 1448)	<.001	0.08

a SPPB: Short Physical Performance Battery.

b Values in parentheses indicate percentage change relative to each subgroup’s baseline mean

## Discussion

### Summary of Key Findings

This study demonstrated that AI-assisted dynamic postural control screening combined with adaptive training significantly improved functional performance among older adults over a 6-month period. Improvements were observed in overall SPPB scores, gait speed, and sit-to-stand performance, with effect sizes ranging from moderate to large (Cohen *d*=0.39‐0.62). Furthermore, the participation rate was positively associated with functional gains, indicating a dose-response relationship.

### Comparison With Prior Work

Our results align with previous AI- and sensor-based fall risk assessment studies [3,5,9,13,18,19]. Importantly, our findings expand on recent *JMIR* research exploring digital health interventions for fall prevention [14,15]. Classic reviews and epidemiological studies have long established that falls among older adults are preventable through multifactorial interventions [20]. In addition, the screening component itself warrants emphasis. Early and objective detection of deficits in mobility, balance, and gait is a prerequisite for timely intervention, particularly as conventional screening tools such as the SPPB are subject to variability and may not scale efficiently in community or clinical settings [4,12]. By embedding AI-based screening in our design, we demonstrated not only intervention efficacy but also the feasibility of scalable, objective, and standardized risk detection, which justifies the inclusion of the screening part of the study. This represents a shift from using AI solely for risk identification toward using it as a tool for delivering individualized, dynamic interventions. While conventional screening tools such as the SPPB are validated, they are limited by interrater variability and scalability issues. Few existing AI interventions have incorporated both real-time skeletal tracking and adaptive exercise prescriptions, making our approach distinct from most prior AI studies. In contrast, our study demonstrates that AI-assisted screening combined with adaptive prescriptions can not only identify deficits but also drive functional improvements in a large older adult cohort. A structured comparison of prior AI applications and this study is summarized in Table 3, highlighting how our work advances beyond assessment toward intervention.

**Table 3.** Comparison of studies using artificial intelligence (AI) and Short Physical Performance Battery (SPPB) to assess physical function in older adults.

Study	Title	Research focus	Methods used	Key findings	Contribution to the field
Friedrich et al [3]	A Deep Learning Approach for TUG^a^ and SPPB Score Prediction of (Pre-) Frail Older Adults on Real-Life IMU Data	Predicting TUG and SPPB scores in prefrail and frail older adults using real-life IMU^b^ data	IMU sensor on the right hip SPPB and TUG tests LSTM^c^+CNN^d^ models Train-validation-test split	TUG prediction accuracy: 95.9% SPPB prediction accuracy: 94.3%	Demonstrates feasibility of predicting physical performance in older adults using real-life IMU data Supports early detection and intervention for physical decline in the older adults
González-Castro et al [5]	The Applications of Artificial Intelligence for Assessing Fall Risk: Systematic Review	Analyzing AI applications in assessing postural control and fall risk	Literature review of 22 studies Accelerometers, video, EHR^e^, and big data Various ML^f^ algorithms (SVM^g^, RF^h^, LSTM, etc)	AI model accuracy: >70% Gait speed and posture are important predictors Accelerometers are the most common data source	Confirms AI as a useful tool for fall risk screening Future focus should be on integrating and validating AI models in clinical practice
Kouno et al [7]	Introduction of AI Technology for Objective Physical Function Assessment	Reviewing current research on AI-based objective physical function assessment	Literature review of 26 studies Video, sensors, EHR, and questionnaires ML regression or classification (SVM, RF, LSTM, etc)	Sensors are the main input for gait speed prediction Video is increasingly used for TUG and gait analysis	Validates AI for predicting SPPB, TUG, gait speed, grip strength, and other physical function indices Recommends incorporating AI-assisted physical function assessment into future clinical routines
Kraus et al [19]	Prediction of Physical Frailty in Orthogeriatric Patients Using Sensor Insole–Based Gait Analysis and Machine Learning Algorithms: Cross-sectional Study	Comparing sensor insoles, questionnaires, and conventional assessments in predicting physical frailty in older patients	57 older adult outpatients Sensor insoles SARC-F^i^ questionnaire SPPB and TUG tests Recursive feature elimination KNN^j^ and RF	Sensor insoles+ML models outperform SARC-F and TUG alone Gait parameters are the most predictive features	Supports integrating sensors and ML into fall risk screening Recommends large-scale validation of this approach
Welch et al [4]	The Short Physical Performance Battery (SPPB): A Quick and Useful Tool for Fall Risk Stratification Among Older Primary Care Patients	Evaluating SPPB as a fall risk stratification tool in older adults and exploring its integration with the STEADI^k^ tool	417 community-dwelling older adults SPPB Fall history Balance confidence Multivariable negative binomial regression models	SPPB total and component scores predict 1-year fall risk In the STEADI-positive group, low SPPB scores are associated with higher fall rates	Supports SPPB as a fall risk stratification tool for older adults SPPB shows promise in complementing STEADI screening but requires large-scale study validation
Marcos-Pardo et al [6]	GDLAM and SPPB batteries for screening sarcopenia in community-dwelling Spanish older adults: Healthy-age network study	Comparing the diagnostic ability of GDLAM^l^ and SPPB batteries to classify sarcopenia	382 community-dwelling older adults GDLAM battery SPPB ROC^m^ curve analysis	GDLAM has moderate to high sensitivity (60.1%‐72.2%) and moderate to very high specificity (57.6%‐90.7%) SPPB lacks ability to classify sarcopenia	GDLAM battery can screen for sarcopenia in community-dwelling older adults GDLAM is more reliable and valid than SPPB for sarcopenia assessment
Duncan et al [18]	Automated Camera-Based Assessment of Short Physical Performance Battery (SPPB) for Older Adults with Cancer	Introducing a camera system for automated SPPB evaluation in older patients with cancer	Motorized camera system SPPB tests Template matching, Haar cascades, and CSRT^n^ tracking In vivo testing with 8 volunteers	Gait speed accuracy: >95% Balance and 5TSS^o^ time accuracy: >97%	Automates SPPB evaluation for older patients with cancer Reliable, efficient, and overcomes limitations of manual assessment
Santos et al [11]	Short Physical Performance Battery (SPPB) score as a discriminator of dynapenic abdominal obesity among community-dwelling older adults	Examining SPPB score as a discriminator of dynapenic abdominal obesity in older adults	382 community-dwelling older adults SPPB Hand grip strength Waist circumference ROC curve analysis	SPPB scores inversely associated with DAO^p^ SPPB cut-off of ≤9 discriminates DAO	SPPB can screen for DAO in older adults SPPB is a useful tool for early DAO detection and management

a TUG: Timed Up and Go.

b IMU: inertial measurement unit.

c LSTM: long short-term memory.

d CNN: convolutional neural network.

e EHR: electronic health record.

f ML: machine learning.

g SVM: support vector machine.

h RF: random forest.

i SARC-F: Simple Questionnaire to Rapidly Diagnose Sarcopenia.

j KNN: k-nearest neighbor.

k STEADI: Stopping Elderly Accidents, Deaths, and Injuries.

l GDLAM: Grupo de Desenvolvimento Latino-Americano para a Maturidade.

m ROC: receiver operating characteristic.

n CSRT: channel and spatial reliability tracking.

o 5TSS: 5 times sit-to-stand.

p DAO: dynapenic abdominal obesity.

### Practical Implications

The findings suggest that AI-assisted systems have potential for broad application in community and clinical settings. By automating data capture and analysis, the AI platform provides objective, real-time, and scalable assessments that reduce reliance on subjective manual scoring. The adaptive prescription framework further allows for individualized exercise programs that can be implemented in day care centers, senior fitness programs, and long-term care facilities. Such systems may enhance efficiency for health care staff while promoting functional independence among older adults.

### Limitations

Several limitations should be noted. First, fall incidence was not directly measured; therefore, conclusions should be interpreted as functional performance improvements rather than confirmed reductions in fall events [21]. Second, the recruitment of the experimental group from community centers and the control group from health care institutions introduced baseline differences in functional status. Although statistical analyses confirmed that these differences did not bias the outcomes, they may limit comparability. Third, the study was conducted in Keelung, Taiwan, which may limit generalizability to populations with different cultural, socioeconomic, or rural backgrounds. Fourth, the follow-up period of 6 months reflects short-term to midterm outcomes; longer-term effects remain unknown.

### Future Research

Future studies should extend follow-up to 12 to 24 months to examine the sustainability of functional gains and to prospectively track fall incidence [22]. Multisite validation across diverse populations, including rural communities, lower-income groups, and culturally varied settings, is required to establish generalizability. Further work should also investigate adherence, engagement, and the accessibility of AI technologies, particularly among populations considered vulnerable who may face barriers to digital health adoption.

### Conclusions

This study demonstrated that AI-assisted dynamic postural control screening, when combined with adaptive training, significantly improved functional mobility among older adults. Improvements were evident in SPPB scores, gait speed, and sit-to-stand performance, with effect sizes in the moderate-to-large range. These results, based on a large sample of more than 2000 participants, provide strong support for the feasibility of implementing AI-assisted programs at scale. Importantly, participation levels showed a dose-response relationship with outcomes, highlighting the role of consistent engagement in maximizing functional benefits.

While the findings are encouraging, several considerations remain. Fall incidence was not directly measured; therefore, conclusions should be interpreted as functional improvements rather than confirmed reductions in fall risk. The 6-month study duration limits insights into long-term sustainability, and the recruitment of participants primarily from community centers and health care institutions in Keelung, Taiwan, may constrain generalizability. Furthermore, the accessibility and usability of AI-based systems for older adults in rural or low-resource settings require further examination.

In summary, this study contributes large-scale evidence that AI-assisted dynamic postural control screening with adaptive training can enhance functional performance in older adults. Future research should extend longitudinal follow-up to 12 to 24 months, prospectively measure fall events, and conduct multisite trials across diverse cultural and socioeconomic contexts. Addressing adherence, technology accessibility, and digital literacy will be essential to ensure equitable adoption and long-term impact of AI-assisted older adult care solutions.

## Abbreviations

AI: artificial intelligence
SPPB: Short Physical Performance Battery
